# Synthesis, Structural and Luminescence Properties of Mn^4+^-Activated Medium-Entropy Perovskites (La_1/3_Gd_1/3_Y_1/3_)_1−*x*_*M_x_*AlO_3−*δ*_ (*M* = Ca, Sr)

**DOI:** 10.3390/molecules31203744

**Published:** 2026-10-18

**Authors:** Akash S. Padole, Vasyl Hreb, Vitalii Stadnik, Yurii Hirskyi, Halyna Zhydachevska, Leonid Vasylechko, Yaroslav Zhydachevskyy

**Affiliations:** 1 Institute of Physics, Polish Academy of Sciences, Aleja Lotników 32/46, 02-668 Warsaw, Poland; padole@ifpan.edu.pl (A.S.P.); hzhydach@ifpan.edu.pl (H.Z.); 2 Lviv Polytechnic National University, S. Bandera Street 12, 79013 Lviv, Ukraine; vasyl.m.hreb@lpnu.ua (V.H.); vitalii.y.stadnik@lpnu.ua (V.S.); yurii.v.hirskyi@lpnu.ua (Y.H.); leonid.o.vasylechko@lpnu.ua (L.V.)

**Keywords:** medium-entropy oxides, Mn^4+^ phosphor, far-red emission, rare-earth aluminate, persistent luminescence, thermally stimulated luminescence, charge compensation, phase composition

## Abstract

Medium-entropy aluminate perovskites of nominal composition (La_1/3_Gd_1/3_Y_1/3_)_1−*x*_*M*_*x*_AlO_3−*δ*_:0.1%Mn^4+^ (*x* = 0, 0.05, 0.1; *M* = Ca, Sr), were prepared by sol-gel and solid-state routes to examine the effects of synthesis, annealing temperature and A-site substitution on their luminescence. In the case of alkaline-earth-free (La_1/3_Gd_1/3_Y_1/3_)AlO_3_ system, single-phase materials are formed after heat treatment of sol-gel derived products at the temperature of 1000–1100 °C. Calcium-substituted samples prepared via the sol-gel method are single-phase over a wide annealing temperature range of 1000–1500 °C, whereas strontium-containing samples contain a minor amount of the secondary-phase RESrAl_3_O_7_. Solid-state products are multiphase materials, containing perovskite and garnet phase, with extra RECaAl_3_O_7_ phase in Ca-substituted samples. The unchanged emission position and spectral profile indicate that Mn^4+^ ions occupy the same octahedral coordination environment of the perovskite structure in all samples. Moreover, no additional spectral features associated with a garnet phase were detected. However, phase composition, microstructure, and defect chemistry affected PL intensity and trapping. For sol-gel samples, PL was strongest at 1450 °C, whereas TSL was strongest at 1100 °C. Ca^2+^ and Sr^2+^ substitution reduced both signals and modified the glow-band shape, with Ca causing the larger decrease, consistent with dopant partitioning and charge compensation. Broad TSL may reflect distributed trap environments from mixed La/Gd/Y occupation. Solid-state samples showed stronger PL, higher TSL, and measurable UV-excited afterglow.

## 1. Introduction

Over the past decade, configurational disorder has become an important design strategy for functional oxides. The concept was first developed for equiatomic metallic alloys [1,2,3] and subsequently carried over to oxide systems [4], since then the number of reported compositions has grown steadily, yielding materials with good mechanical strength, thermal stability and resistance to demanding environments [5,6]. Such oxides are classified by their configurational entropy, *S*_config_ = −*R*∑_i_ *x*_i_ln*x*_i_, where *x*_i_ is the mole fraction of the *i*-th cation on the mixed site [5,7]. Values above 1.5*R* define a high-entropy composition, whereas the interval from 1*R* to 1.5*R* corresponds to the medium-entropy range [8,9]. Three cations in equal amounts on one sublattice, therefore, give *R* ln 3 ≈ 1.10*R*. These limits are conventional rather than physical, and the value obtained depends on whether the summation is taken over the entire structure or over the individual sublattices [7,8,9,10].

Perovskites suit this approach well, since the ABO_3_ framework accommodates substitution on either cation site without losing structural integrity [11]. Accordingly, high-entropy perovskites have been reported for oxygen evolution [12], energy storage [13], proton conduction [14], and oxygen transport [15]. Medium-entropy compositions have been prepared for several of the same purposes [16,17,18], and are of interest because entropy stabilisation does not in fact require reaching the 1.5*R* threshold [9]. For a phosphor the useful feature is that the two cation sites can be treated separately. An activator on the octahedral B site is surrounded only by oxygen, so its nearest neighbours are the same in every composition, whatever cations occupy the A site. The mixed cations lie one shell further out and act on the activator indirectly, through the octahedral tilting and local strain that follow from their differing sizes [19,20]. A-site disorder should therefore alter the wider environment and the defect chemistry more than the crystal field at the emitting ion. Cation disorder has been used in phosphor design before, but mainly to enhance the emission itself [21,22].

Among the available activators, Mn^4+^ is well suited to such a host [23]. In conventional white LEDs, a blue chip combined with a YAG:Ce^3+^ phosphor emits insufficiently in the long-wavelength region, which leaves the correlated colour temperature high and the colour rendering poor [23]. Mn^4+^ in octahedral coordination emits in exactly this region, giving a narrow band in the red or far red from the spin-forbidden ^2^E_g_ → ^4^A_2g_ transition, which is also useful for plant growth lighting [24,25]. Rare-earth aluminate perovskites are suitable hosts, being structurally stable, wide in band gap, and able to accept different rare-earth cations [26,27]. Their distorted structure favours intrinsic defects such as oxygen and cation vacancies, antisite defects, and defect complexes, which trap charge and thereby control thermally stimulated luminescence (TSL) and persistent luminescence (PersL) [26,28]. The two properties therefore have different origins: the Mn^4+^ emission depends on the local crystal field [29,30], whereas the TSL and the PersL depend on the traps and on their depth and stability [28].

Substitution is the usual way of adjusting this defect chemistry. When Ca^2+^ or Sr^2+^ replaces a trivalent rare earth, the charge balance is disrupted and compensating defects are formed [31]. These may trap carriers and give rise to the TSL and the PersL [32], or create non-radiative pathways that reduce the emission efficiency, depending on which defects form and in what concentration [33]. In LaAlO_3_:Mn^4+^, co-doping with Ca^2+^ or Sr^2+^ has been reported to enhance both the photoluminescence and the PersL [31,32], and in *R*AlO_3_ phosphors in general, the trap-related emission follows the intrinsic defects that release the carriers [26]. A-site mixing has been studied separately, in Y_1−*x*_Gd*_x_*AlO_3_:Mn^4+^ and Gd_1−γ_La_γ_AlO_3_:Mn^4+^ solid solutions, where the trap depths decrease along the cation sequence Y → Gd → La [26]. That work is isovalent, however, so the charge balance remains intact. The same applies to isovalent A-site substitution in undoped aluminate perovskites, where replacing one rare earth by another alters the lattice parameters and the optical response without introducing compensating defects [34]. A multi-cation A site has recently been shown to form in this structure [35], but its effect on the trap structure has not been examined.

We used three rare earths on the A site. Each Mn^4+^ ion then sits in a slightly different environment, so the crystal-field strength varies from site to site, while the composition stays simple enough that any change in behaviour can still be traced to a particular substitution. Samples of (La_1/3_Gd_1/3_Y_1/3_)_1−*x*_*M_x_*AlO_3−*δ*_:0.1%Mn^4+^ with *x* = 0, 0.05 and 0.1 and *M* = Ca or Sr were prepared by sol-gel and solid-state routes (see Section 3). 

The configurational entropy per mole of A-site cations is 1.10*R*, 1.24*R* and 1.31*R*, so all compositions fall in the medium-entropy range. Our aim was to find how the annealing temperature, the divalent substitution and the synthesis route affect the Mn^4+^ photoluminescence, the TSL and the PersL, and whether disorder on the A site shows up in the trap structure at all. 

## 2. Results and Discussion

### 2.1. Phase Formation and Crystal Structure

**Figure 1:**
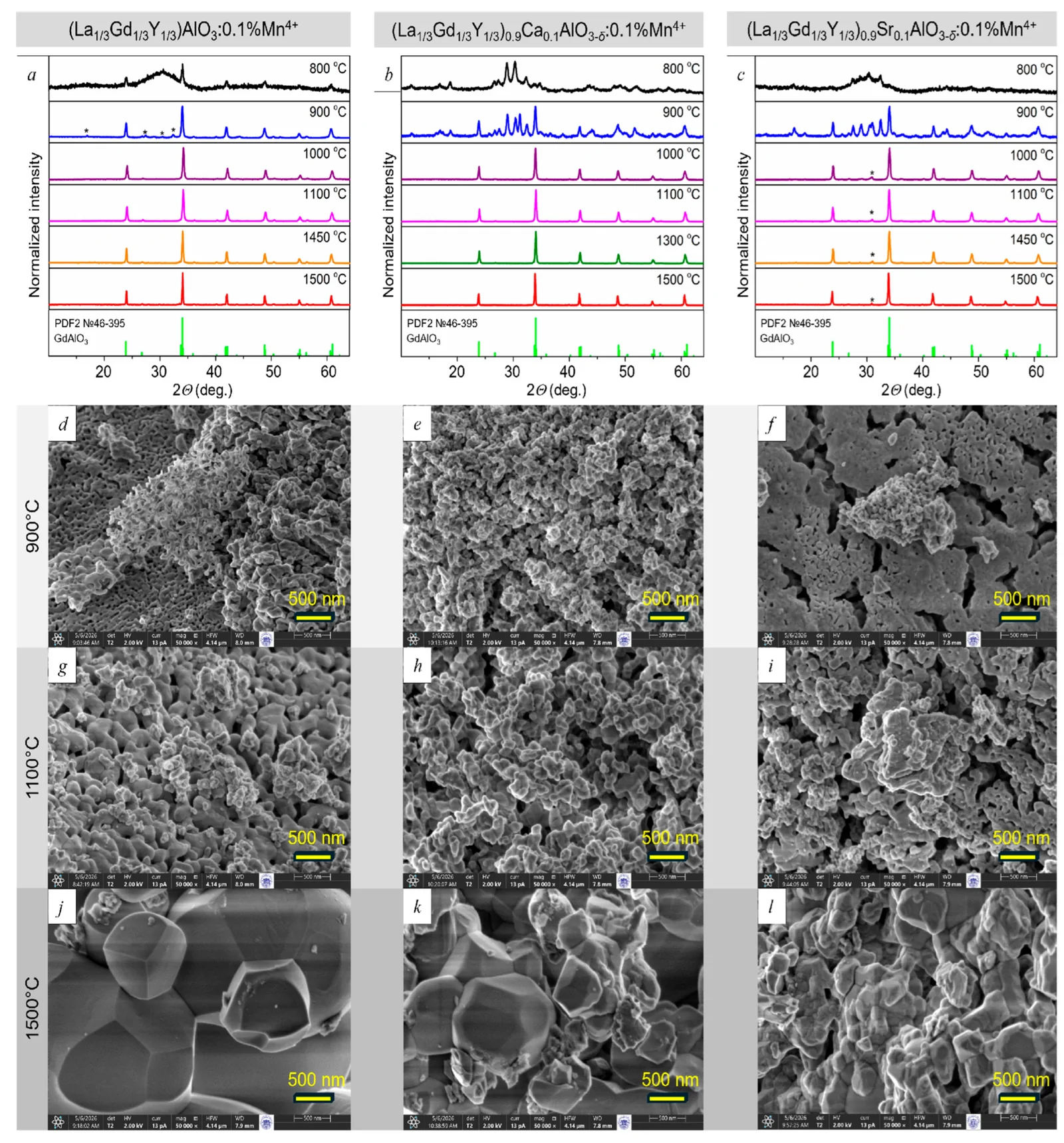
Experimental XRD patterns (**a**–**c**); asterisks (*) mark diffraction peaks assigned to the garnet and other secondary phases. SEM images (**d**–**l**) of LGYAO:Mn, LGYAO:Mn,Ca_0.10_, and LGYAO:Mn,Sr_0.10_ powder samples heat-treated at different temperatures.

Simultaneous DTA/TG combined with XRD showed that the sol-gel LGYAO:Mn material begins to crystallise near 870 °C. After 4 h at 900 °C the sample was already mostly perovskite, with only a small amount of a secondary phase remaining (Figure 1a, left panel). Stepwise annealing at 1000, 1100, 1450 and 1500 °C then improved the phase purity and the structural order, as the secondary phase disappeared and the perovskite Bragg reflections narrowed (Figure 1a). The powders annealed at 1000 and 1100 °C were single-phase perovskite, but about 1% of a garnet phase appeared again after 1450 and 1500 °C. Note that similar precipitation of the yttrium-containing garnet phase was recently observed in the case of five-component high-entropy perovskite (Y_0.2_Nd_0.2_Sm_0.2_Eu_0.2_Er_0.2_)AlO_3_ after heat treatment of sol-gel-derived product above 1200 °C [35]. SEM analysis shows that the 900 °C LGYAO:Mn powder is nanocrystalline, with particles of 20–50 nm; these grow to 100–200 nm at 1100 °C (Figure 1d,g) and, after 5 h at 1500 °C, to 1–2 μm (Figure 1j).

In the Ca- and Sr-substituted compositions LGYAO,Ca and LGYAO,Sr, several crystalline phases were present after calcination of the powders at 900 °C (Figure 1b,c). Annealing at 1000 °C gave phase-pure LGYAO,Ca_0.1_ material with a perovskite structure, and this phase remains stable up to the highest annealing temperature of 1500 °C. In the Sr-containing LGYAO,Sr_0.1_ sample, the materials annealed between 1000 and 1500 °C consisted mainly of the perovskite phase together with a detectable amount of the tetragonal RESrAl_3_O_7_ phase (ICDD PDF-2 cards No. 50-1815, 50-1817 and 49-604 for LaSrAl_3_O_7_, GdSrAl_3_O_7_ and YSrAl_3_O_7_ respectively) (Figure 1c). The appearance of this secondary phase in the (La_1/3_Gd_1/3_Y_1/3_)_0.90_Sr_0.10_AlO_3−*δ*_ materials, marked with asterisks in Figure 1c, shows that the solubility of Sr in the (LaGdY)AlO_3_ perovskite lattice is limited. The crystallinity of the three series of the LGYAO samples under investigation increased progressively with stepwise annealing up to 1500 °C. Particle growth, however, is less pronounced in the Ca- and Sr-substituted samples than in the unsubstituted material as can be seen by comparing Figure 1j,k,l.

**Figure 2:**
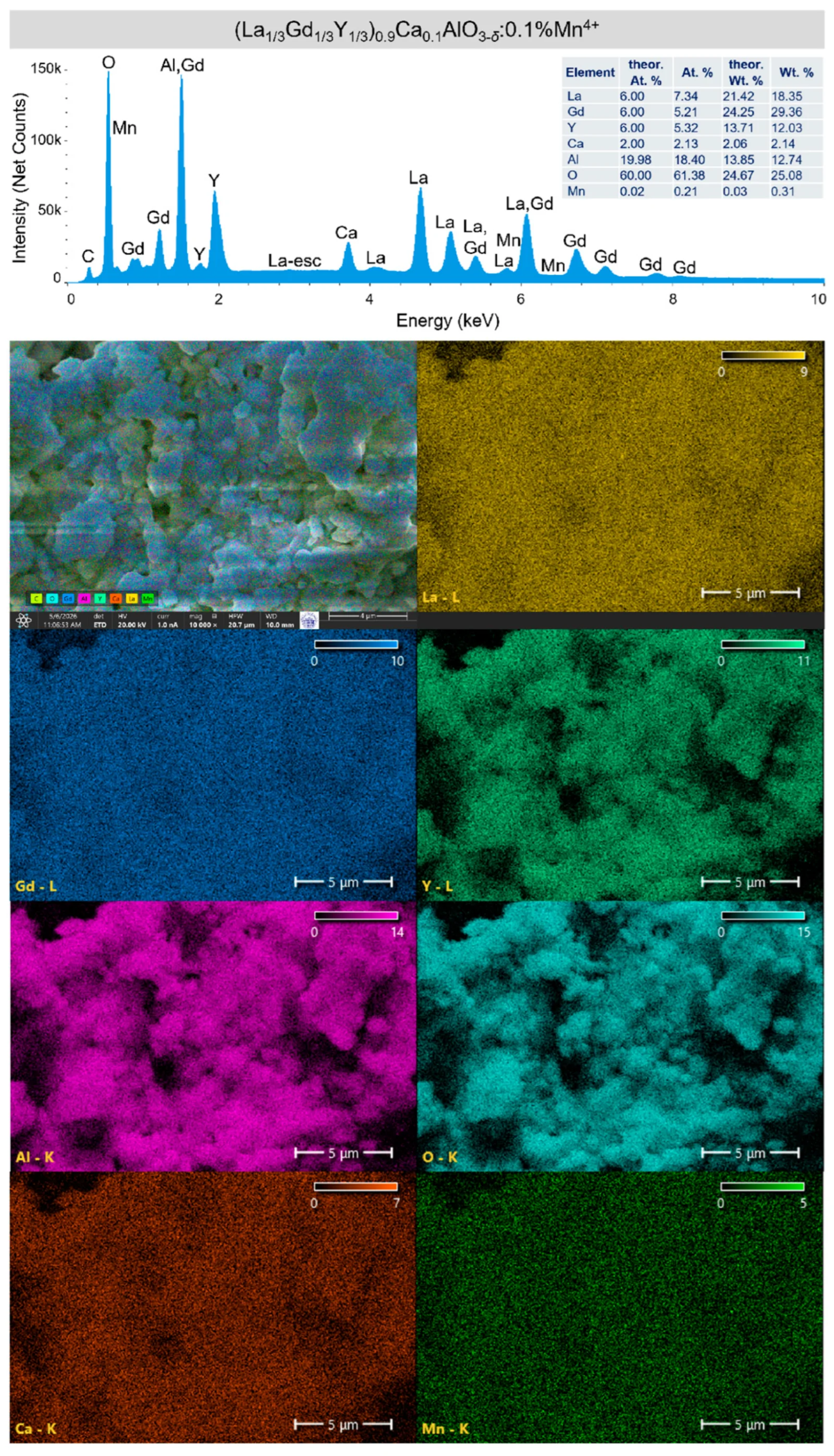
EDX survey spectrum, elemental composition, and elemental distribution maps of the matrix and dopant elements in the sol-gel-derived material with the nominal composition (La_1/3_Gd_1/3_Y_1/3_)_1−*x*_Ca_*x*_AlO_3−*δ*_:0.1%Mn^4+^, annealed at 1500 °C.

EDX analysis of the three-sample series showed that the measured compositions agreed with the nominal ones within the experimental error. Elemental mapping confirmed that La, Gd, Y, Al and O, as well as Ca, Sr and Mn, were distributed uniformly over the powder particles. Representative EDX results are shown in Figure 2.

**Figure 3:**
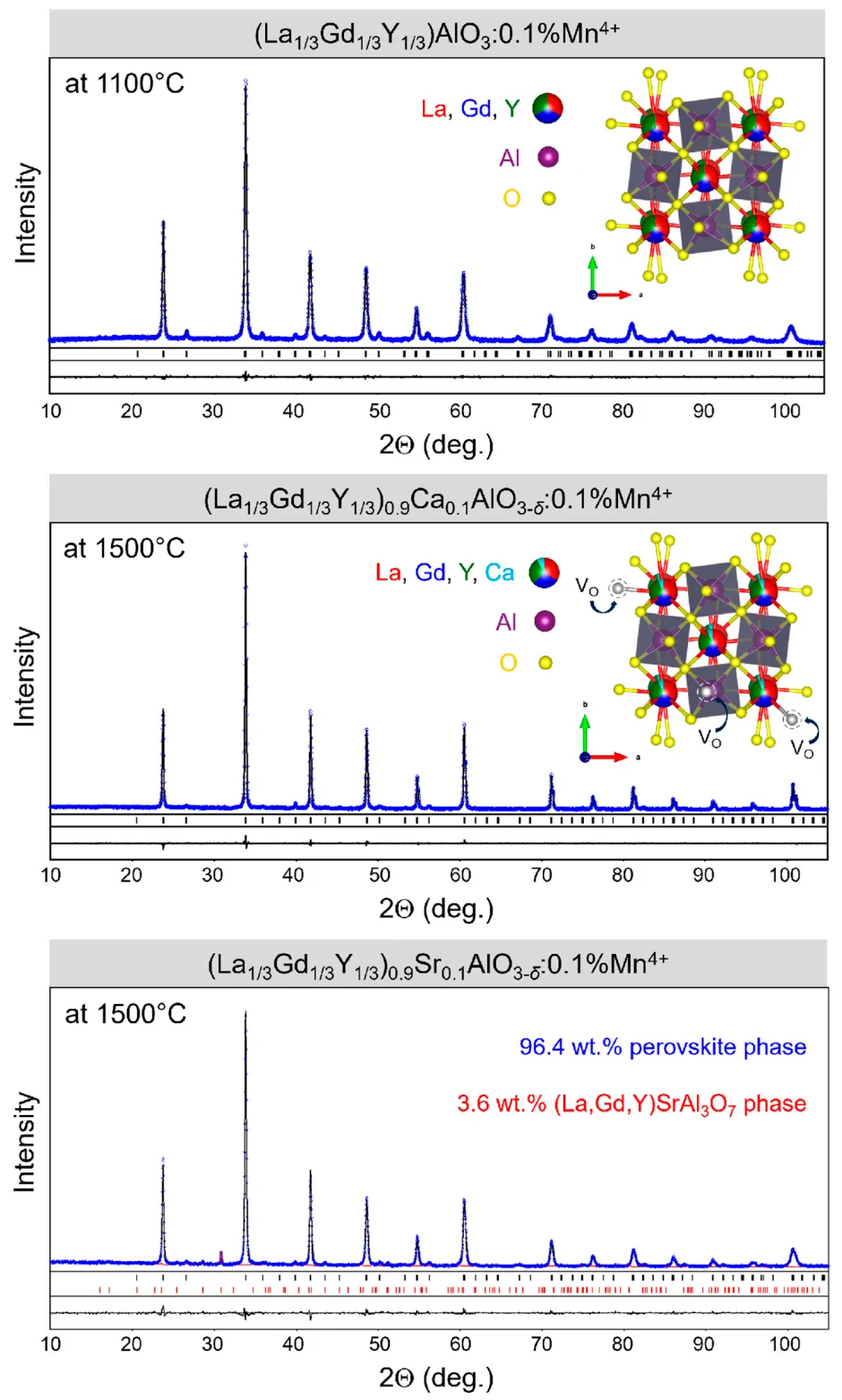
Graphical results of Rietveld refinement of (La_1/3_Gd_1/3_Y_1/3_)AlO_3_ (**top**), (La_1/3_Gd_1/3_Y_1/3_)_0.9_Ca_0.1_AlO_3−*δ*_ (**middle**) and (La_1/3_Gd_1/3_Y_1/3_)_0.9_Sr_0.1_AlO_3−*δ*_ (**bottom**) materials annealed at 1100 °C and 1500 °C. Experimental XRD patterns (small blue circles) are shown together with calculated diffraction profiles and difference curves between them (black lines). Short vertical bars indicate the positions of Bragg’s maxima in the orthorhombic *Pbnm* structure of the main perovskite phase (black marks) and admixture tetragonal RESrAl_3_O_7_ phase (red marks). The insets show a Vesta representation of the structures as a three-dimensional grid of corner-shared AlO_6_ octahedra, with RE^3+^, Ca^2+^ and Sr^2+^ ions statistically distributed in the holes between them.

Crystal structure parameters of the synthesised materials were evaluated from experimental XRD data by full profile Rietveld refinement using the WinCSD programme package, version 4.19 [36]. The unit cell dimensions, the atomic coordinates and the displacement parameters were refined for all atoms after adjusting of profile parameters and corrections for absorption and instrumental sample shift. For the single-phase materials (La_1/3_Gd_1/3_Y_1/3_)AlO_3_ and (La_1/3_Gd_1/3_Y_1/3_)_0.9_Ca_0.1_AlO_3−*δ*_, excellent agreement between the calculated and experimental XRD profiles was achieved during the structure refinement in the *Pbnm* space group using the atomic coordinates in the GdAlO_3_ structure [27] as the initial model (see Figure 3, top and middle panel). These patterns clearly confirm the phase purity and orthorhombic perovskite structure of both samples. The refined structural parameters of (La_1/3_Gd_1/3_Y_1/3_)AlO_3_ and (La_1/3_Gd_1/3_Y_1/3_)_0.9_Ca_0.1_AlO_3−*δ*_ materials are collected in Table 1. For the stoichiometric composition, no deviation from the nominal composition was found, whereas for the aliovalently substituted (La_1/3_Gd_1/3_Y_1/3_)_0.9_Ca_0.1_AlO_3−*δ*_ material, a small deficiency was detected at the O1 position in the 4c Wyckoff site (Table 1 and the inset in the middle panel of Figure 3).

For the Sr-containing material containing admixtures tetragonal phase, a simultaneous two-phase Rietveld refinement was performed. To model the X-ray diffraction profile of the tetragonal phase, atomic coordinates from the YSrAl_3_O_7_ structure (space group *P*-42_1_*m*) [37] were used. During the refinement process, only the unit cell parameters of the minor tetragonal phase were refined, whereas a complete set of structural parameters was obtained for the main perovskite phase, as listed in Table 1.

Graphical results of Rietveld refinement proving a presence of 3.6 wt.% of tetragonal phase in the sample of nominal composition (La_1/3_Gd_1/3_Y_1/3_)_0.9_Sr_0.1_AlO_3−*δ*_ are presented on the bottom panel of Figure 3. Based on a comparison of the refined lattice parameters of the secondary tetragonal phase formed in the (La_1/3_Gd_1/3_Y_1/3_)_0.9_Sr_0.1_AlO_3−*δ*_ material (*a* = 7.833(2) Å, *c* = 5.167(2) Å, *V* = 317.0(2) Å^3^) with literature data for the individual compounds LaSrAl_3_O_7_, GdSrAl_3_O_7,_ and YSrAl_3_O_7_ [37] it can be concluded that this phase contains all three RE elements in approximately equal proportions, and its composition can be written as (La,Gd,Y)SrAl_3_O_7_.

A comparison of the refined structural parameters of the materials presented in Table 1 indicates that the substitution of RE^3+^ cations in (La_1/3_Gd_1/3_Y_1/3_)AlO_3_ structure with Ca^2+^ affects the lattice dimensions in opposite ways. The *a* parameter of the Ca-substituted material is larger than that of the unsubstituted one, whereas the *b* and *c* parameters are smaller, which results in a measurable decrease in the unit cell volume (Table 1). Consequently, the orthorhombic distortion, defined as *b* − *a*, decreases from 0.0207 Å to 0.0040 Å. In eight-fold coordination, the ionic radius of Ca^2+^ (1.12 Å) is larger than the mean radius of La^3+^, Gd^3+^ and Y^3+^ cations (1.08 Å) [38], and a larger A-site cation raises the tolerance factor, thereby diminishing the orthorhombic distortion of the perovskite structure. The slight decrease in unit-cell volume can be attributed to oxygen deficiency, revealed by structural refinement.

Similar opposing changes in the orthorhombic perovskite lattice parameters are also observed for the (La_1/3_Gd_1/3_Y_1/3_)_0.9_Sr_0.1_AlO_3−*δ*_ structure (see Table 1), the distinction is that in the strontium-doped material, aliovalent substitution resulted in an increase in two lattice parameters—*a* and *c*—alongside a moderate contraction of parameter *b*. Accordingly, the unit cell volume of (La_1/3_Gd_1/3_Y_1/3_)_0.9_Sr_0.1_AlO_3−*δ*_ is larger than that of the Sr-free material, due to the larger ionic radius of Sr^2+^ (1.26 Å) compared with the radii of the constituent RE cations. These observations, along with the above-mentioned results of phase analysis regarding precipitation of the secondary Sr-containing phase, indicate that the solubility limit of strontium in the medium-entropy perovskite (La_1/3_Gd_1/3_Y_1/3_)_1−*x*_Sr_*x*_AlO_3−*δ*_ is slightly less than *x* = 0.1.

**Figure 4:**
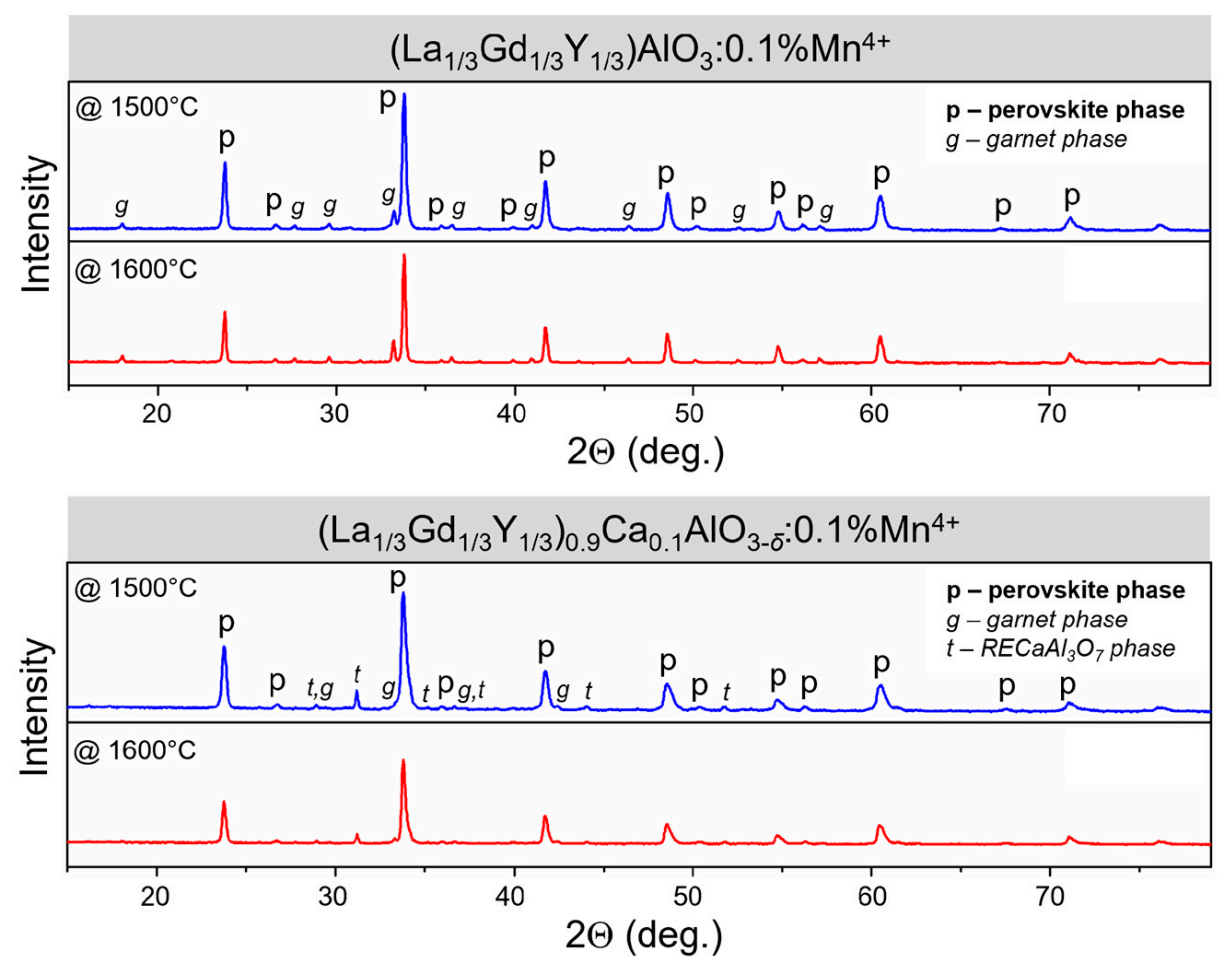
Experimental XRD patterns of LGYAO and LGYAO,Ca_0.1_ samples prepared by the solid-state reaction method at 1500 °C and subsequently annealed at 1600 °C. The blue and red lines correspond to the patterns recorded after heat treatment at 1500 and 1600 °C, respectively.

In contrast to the sol-gel route, the solid-state synthesis did not give phase-pure materials. For the alkaline-earth-free LGYAO sample, annealing at 1500 °C produced a mixture of the main perovskite phase and a secondary garnet phase, which amount slightly increase after further heat treatment at 1600 °C (Figure 4, top panel). The crystallinity of the powder nevertheless increased, as seen from the narrowing of the perovskite Bragg reflections. In the diffractograms of calcium-substituted (La_1/3_Gd_1/3_Y_1/3_)_0.9_Ca_0.1_AlO_3−*δ*_ sample prepared via solid-state synthesis at 1500 °C, in addition to the above-mentioned garnet impurity, significant amount of secondary tetragonal RECaAl_3_O_7_ phase (ICDD PDF-2 card No. 50-1808 for GdCaAl_3_O_7_) was detected alongside the main perovskite phase (Figure 4, bottom panel). The relative fraction of this parasitic phase decreases by almost half after additional annealing at 1600 °C; however, its residual peaks are clearly detectable on the diffractogram. These results suggest that the incorporation of such an amount of calcium into the LGYAO perovskite lattice is doubtful when the material is prepared by solid-state reaction.

### 2.2. PL and PLE Spectral Features and Decay Kinetics

**Figure 5:**
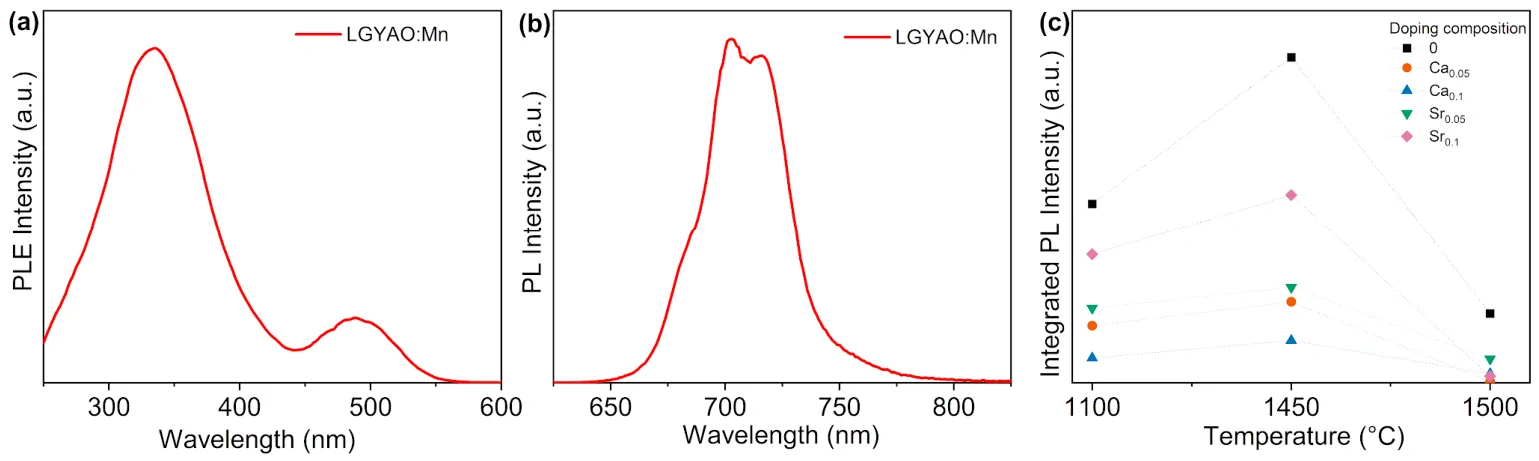
PL/PLE spectra and integrated PL intensity of sol-gel-derived samples. (**a**) PLE spectrum of LGYAO:Mn annealed at 1450 °C, monitored at around 700 nm. (**b**) PL spectrum recorded under 490 nm excitation, showing the characteristic Mn^4+^ red emission around 700 nm. (**c**) Integrated PL intensity of sol-gel-derived samples annealed at 1450 °C from the baseline-corrected Mn^4+^ emission band.

Representative PL and PLE spectra of the unsubstituted LGYAO:Mn sample annealed at 1450 °C are shown in Figure 5a,b. The emission was recorded under 490 nm excitation and consists of a single red band centred near 700 nm, which is the position expected for Mn^4+^ in an octahedral crystal field. It is assigned to the spin-forbidden ^2^Eg → ^4^A_2g_ transition. This assignment is consistent with the Rietveld refinement in Section 2.1, in which Mn^4+^ was placed on the octahedrally coordinated Al 4b site (Table 1).

The PLE spectrum was measured for the 700 nm emission and shows two bands in the covered range. The weaker band near 490 nm, is assigned to the ^4^A_2g_ →^4^T_2g_ transition of Mn^4+^. The stronger band near 335 nm is assigned mainly to O → Mn charge transfer, with an overlapping contribution from the ^4^A_2g_ → ^4^T_1g_ transition [24,26,30]. Together with the red emission, these bands show that Mn^4+^ is the optically active centre in the medium-entropy aluminate perovskite host. A comparable spectrum was obtained for the phase-pure powder annealed at 1100 °C. The emission therefore comes from Mn^4+^ in the perovskite phase; any contribution from the minor garnet phase present after treatment at 1450 and 1500 °C is not resolved in the spectra. The position and overall profile of the emission band remain nearly unchanged throughout the series. Within the resolution of the measurements, the dominant Mn^4+^ ions, therefore, experience a similar octahedral ligand field in all samples, although small changes in local distortion cannot be excluded. The spectral similarity does not mean that the samples are otherwise optically equivalent. Phase composition, crystallinity, particle size, and defect chemistry can change the fraction of optically active Mn^4+^ ions and the competition between radiative and non-radiative relaxation. The same factors can alter the concentration, distribution and filling of charge traps without producing a measurable shift in the Mn^4+^ emission band. Thus, the emitting centre remains the same, whereas the PL intensity, TSL response and afterglow depend strongly on how the material is prepared and substituted.

**Figure 6:**
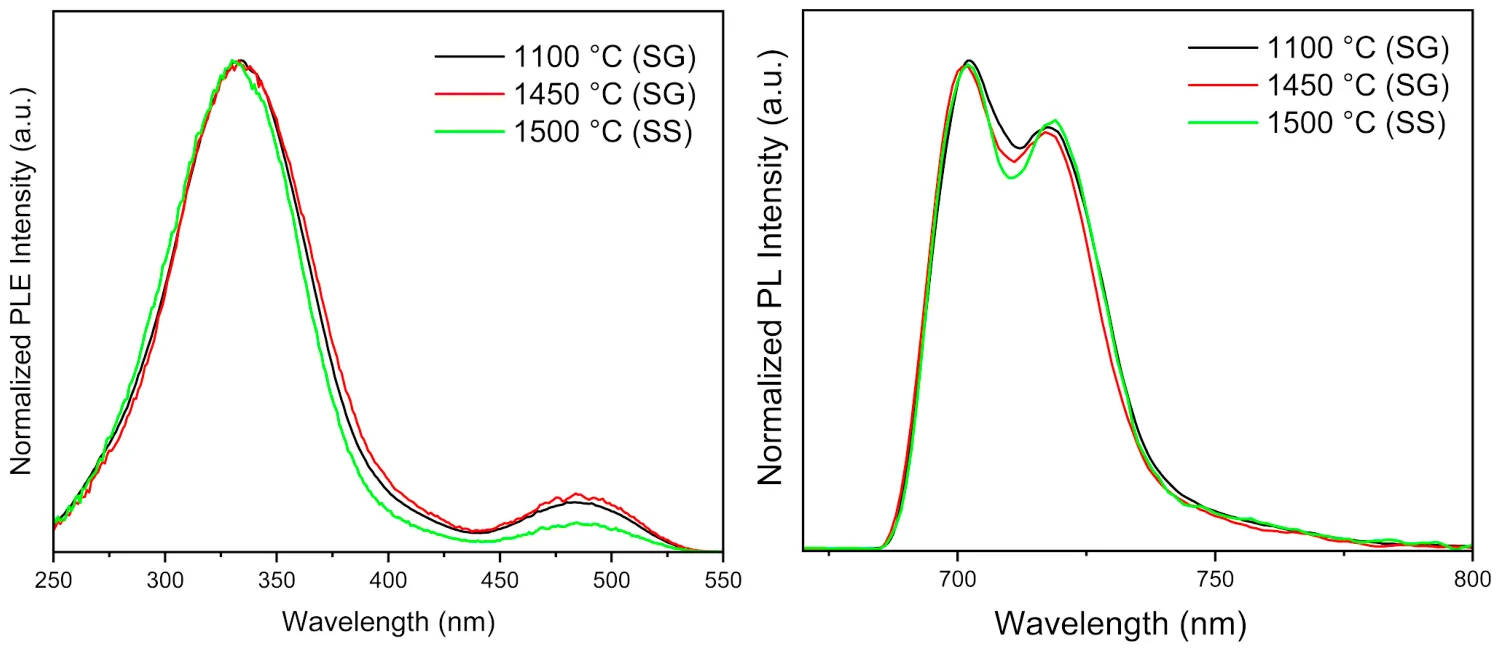
Low-temperature (4.4 K) PL and PLE spectra of LGYAO sol-gel powders annealed at 1100 and 1450 °C, together with the corresponding solid-state sample at 1500 °C. Each spectrum is normalised to its respective maximum intensity.

The PL spectra measured at low temperature (4.4 K) for the phase-pure sol-gel powder annealed at 1100 °C, the sol-gel powder annealed at 1450 °C containing a minor garnet phase, and the solid-state powder containing both garnet and perovskite phases exhibit similar main emission bands near 700 nm (Figure 6). The measured spectra were compared with published PL spectra of YAG:Mn^4+^ [30]; however, no characteristic garnet-related Mn^4+^ emission was detected in our samples. This comparison supports a dominant contribution from the perovskite phase and is consistent with the assignment of the observed emission to Mn^4+^ ions in the perovskite structure.

**Figure 7:**
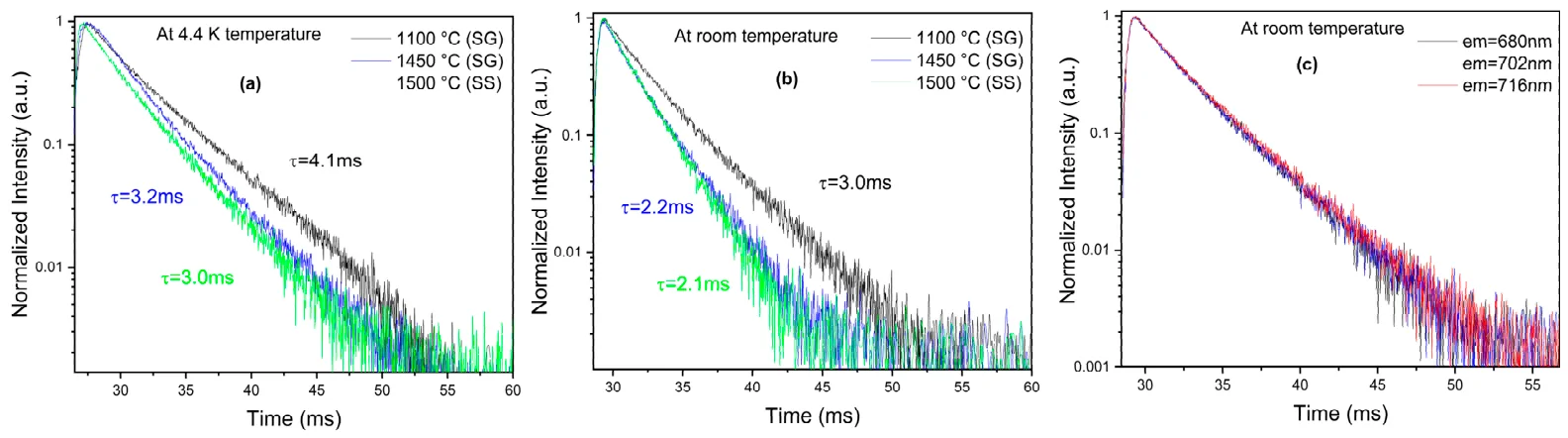
Photoluminescence (PL) decay curves of LGYAO samples, comparing the phase-pure sol-gel sample annealed at 1100 °C with garnet-containing samples prepared by the sol-gel method at 1450 °C and the solid-state method at 1500 °C. The decay kinetics were measured under 490 nm excitation, with emission monitored at 702 nm at (**a**) 4.4 K and (**b**) room temperature. (**c**) PL decay curves of the sol-gel-prepared LGYAO sample annealed at 1100 °C, recorded at room temperature under 490 nm excitation and monitored at different emission wavelengths.

The photoluminescence decay kinetics of the LGYAO:Mn samples, recorded at 4.4 K and room temperature, are presented in Figure 7. All decay curves can be well enough described by a single-exponential function, confirming a dominant presence of a single active emitting centre in the investigated systems.

As summarised in Table 2, the luminescence lifetime varies systematically with the phase composition and preparation method. At 4.4 K, the phase-pure sample annealed at 1100 °C exhibits the largest lifetime of 4.1 ms. However, the emergence of a secondary garnet phase leads to a reduction in the decay time, dropping to 3.2 ms for the sol-gel sample annealed at 1450 °C and further decreasing to 3.0 ms for the solid-state sample prepared at 1500 °C. This progressive shortening of the luminescence lifetime indicates a higher non-radiative transition rate. One can suggest that the secondary garnet impurity phase introduces interfacial boundaries and structural defects that function as effective quenching centres. Figure 7b presents the photoluminescence (PL) decay curves measured at room temperature. The lifetimes follow the same trend as those measured at 4.4 K (Table 2), suggesting that the observed differences in decay dynamics persist across the two temperature conditions. Furthermore, the similar decay kinetics observed at different emission wavelengths (Figure 7c) suggest that the emission is dominated by the same luminescent centre. Trace Mn from starting materials can also produce red emission, as reported for Mg_7_Ga_2_GeO_12_:Tb^3+^ [39]. To test this contribution in the present samples, a host prepared without added Mn under the same conditions is needed; no such control result is included here.

### 2.3. Effect of Annealing Temperature on PL Intensity of Sol-Gel Samples

The effect of the annealing temperature on the Mn^4+^ emission was evaluated from the integrated PL intensity of the sol-gel samples. Since neither the shape nor the position of the emission band changes noticeably from one sample to another, the comparison was based on the integrated intensity of the Mn^4+^ band, taken over the same spectral range in all cases. The result depends strongly on the annealing temperature, as shown in Figure 5c. All sol-gel compositions emit most strongly after treatment at 1450 °C. The samples annealed at 1100 °C are weaker, and a further step to 1500 °C causes a clear drop, so that the order is 1450 > 1100 > 1500 °C.

The increase in PL intensity from 1100 to 1450 °C is consistent with the improvement in crystallinity observed by XRD and the particle growth observed by SEM (Section 2.1). The reduced relative contribution of surfaces, grain boundaries, and disordered regions may decrease non-radiative losses and thereby enhance the emission efficiency of Mn^4+^. However, although the particles continue to grow at 1500 °C, the PL intensity decreases. This indicates that particle growth and improved crystallinity alone cannot fully account for the luminescence behaviour at the highest annealing temperature. The decrease in PL intensity after annealing at 1500 °C may be associated with enhanced non-radiative relaxation. This interpretation is supported by the observed decrease in PL lifetime with increasing annealing temperature, which is consistent with an increase in the non-radiative decay rate. In addition, changes in the secondary-phase content, or defect structure during high-temperature annealing may contribute to the observed quenching. The essentially unchanged emission profile suggests that the dominant Mn^4+^ emitting centre remains unchanged. Among the annealing conditions investigated, the sample annealed at 1450 °C exhibits the highest integrated PL intensity.

### 2.4. Effect of Ca^2+^and Sr^2+^ Substitution on PL Intensity

The effect of Ca^2+^ and Sr^2+^ substitution on the Mn^4+^ emission was studied from the integrated PL intensity of the sol-gel samples annealed at 1450 °C (Figure 5c). The unsubstituted LGYAO:Mn material emits most strongly of all the compositions studied. Substitution with either cation lowers the intensity, so neither of them improves the direct Mn^4+^ emission in this series. Among the substituted samples, the Sr-containing compositions stay brighter than the Ca-containing ones. Sr_0.05_ gives the strongest emission within the substituted series and Ca_0.10_ the weakest, showing that both the identity and concentration of the substituting ion influence the PL intensity.

Neither the position nor the shape of the band changes as the intensity falls, providing no evidence for a second resolved Mn^4+^ emitting centre. The decrease instead reflects a change in the population or efficiency of the dominant centre. Charge compensation provides a possible explanation. Mn^4+^ on an Al^3+^ site has a positive effective charge, whereas Ca^2+^ or Sr^2+^ on a rare-earth site has a negative effective charge and can therefore contribute to compensation. Because the divalent-ion concentration is much higher than the Mn concentration, other compensating processes must also occur. These may include formation of oxygen vacancies, changes in Mn valence, or other point defects. Such changes can affect non-radiative recombination and the fraction of optically active Mn^4+^ ions [33]. The stronger effect of calcium agrees with the phase analysis in Section 2.1: the Ca-substituted sol-gel material is phase pure, so within the detection limit of XRD essentially all the calcium enters the perovskite lattice, whereas part of the strontium is taken by the tetragonal YSrAl_3_O_7_ phase and only the rest is available for compensation. For the sol-gel series, then, the unsubstituted composition gives the most favourable conditions for the direct Mn^4+^ photoluminescence, and both Ca^2+^ and Sr^2+^ lower the emission efficiency.

### 2.5. Comparison of PL Intensity for Sol-Gel and Solid-State Samples

**Figure 8:**
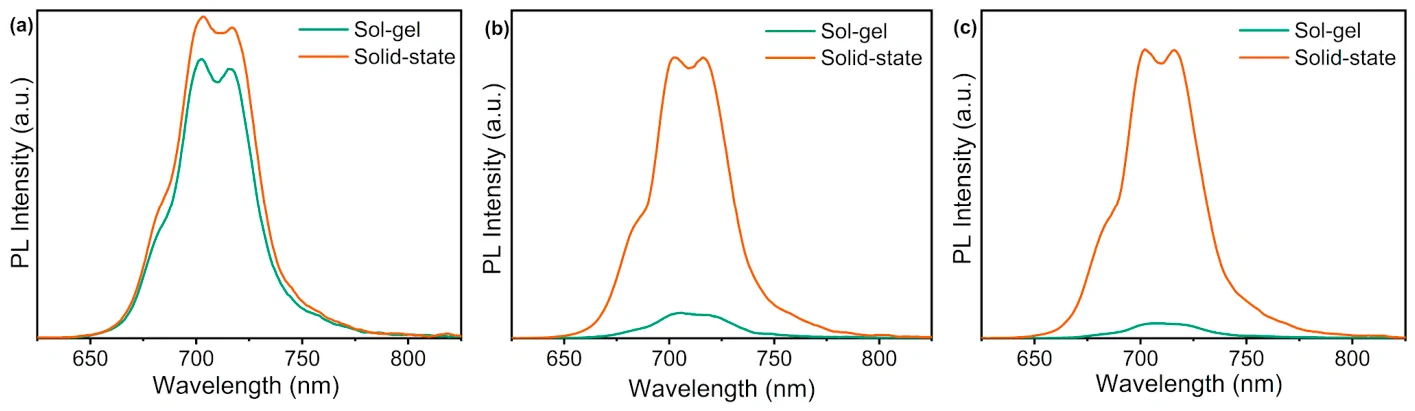
Comparison of the PL intensity of Mn^4+^-doped medium-entropy aluminate samples. (**a**–**c**) PL spectra of sol-gel (SG) and solid-state (SS) samples with the same nominal compositions: LGYAO:Mn, LGYAO:Mn,Ca_0.05_, and LeGYAO:Mn,Ca_0.10_.

The role of the preparation route was studied by comparing sol-gel and solid-state samples of the same nominal composition, recorded under identical excitation and detection conditions (Figure 8a–c). In every case, the solid-state sample emitted more strongly than the sol-gel one. The difference is small for the unsubstituted LGYAO:Mn, larger for Ca_0.05_ and largest for Ca_0.1_. The stronger emission from the solid-state samples probably results from more than one structural difference. Their narrower perovskite reflections and larger particles indicate higher crystallinity and a smaller relative contribution from surfaces and grain boundaries, which may reduce non-radiative losses. Phase composition, particle size, crystallinity, defects and dopant partitioning differed at the same time, so the higher PL cannot be attributed to the synthesis method alone.

The Ca-containing comparison is more complicated because the solid-state powders contain garnet and tetragonal RECaAl_3_O_7_ in addition to the perovskite phase, whereas the corresponding sol-gel powders are single-phase. The two routes therefore differ in phase composition, microstructure and Ca partitioning. The presence of RECaAl_3_O_7_ confirms that at least part of the Ca lies outside the perovskite phase in the solid-state products. Powder XRD, however, cannot determine how much Ca remains in the perovskite or which crystallographic site it occupies. The growing difference in PL intensity with increasing nominal Ca content is compatible with the combined effects of microstructure, phase partitioning and defect compensation, but it does not establish which contribution is dominant. These effects cannot be separated using the present data.

### 2.6. Thermally Stimulated Luminescence

**Figure 9:**
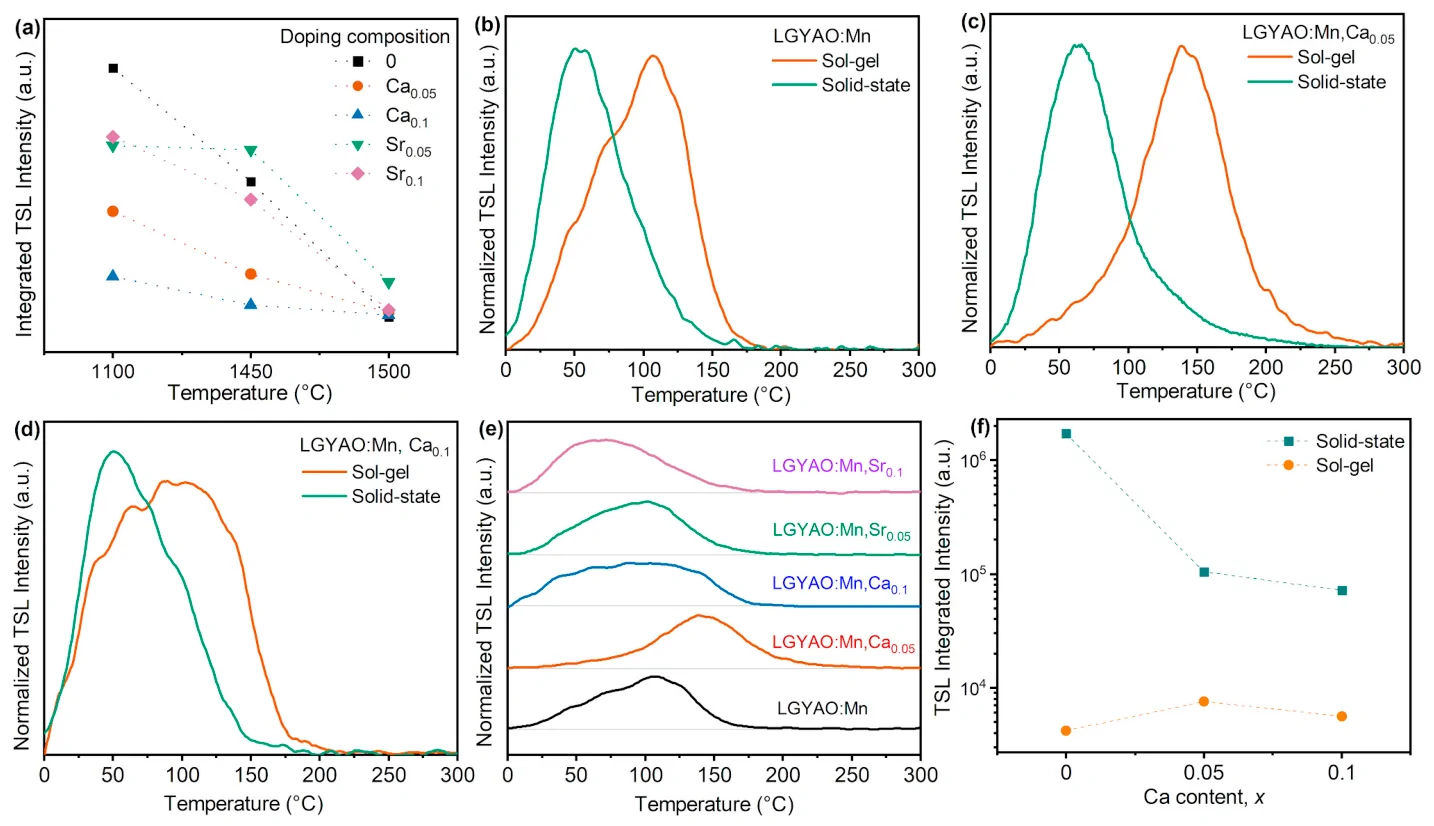
TSL behaviour of the Mn^4+^-doped medium-entropy aluminate samples. (**a**) Integrated TSL intensity of the sol-gel samples as a function of annealing temperature. (**b**–**d**) Normalised TSL glow curves of the sol-gel and solid-state samples: LGYAO:Mn (**b**), LGYAO:Mn,Ca_0.05_ (**c**) and LGYAO:Mn,Ca_0.1_ (**d**); each curve is normalised to its own maximum. (**e**) Normalised TSL glow curves of the sol-gel samples annealed at 1450 °C. (**f**) Integrated TSL intensity of the sol-gel and solid-state samples plotted on a logarithmic scale; intensities were integrated over 0–300 °C and the dotted lines are guides to the eye. All measurements were performed after irradiation at 300 nm for 5 min, with a heating rate of 1 °C s^−1^.

Thermally stimulated luminescence was measured after irradiation at 300 nm for 5 min at 0 °C, followed by heating from 0 to 300 °C at 1 °C/s. Irradiation near 490 nm gave no reliable response under the same conditions (Figure 9). As previously established for the YAlO_3_ host, Mn^4+^ ions can undergo photoionisation via a two-photon absorption process under 490 nm excitation into the first excited level (^4^T_2_) [30,40,41,42]. However, in the mixed *R*AlO_3_ perovskites investigated in this work, this two-photon ionization pathway at 490 nm and a trapped-charge population under such conditions are negligible. This feature requires separate studies, which are out of the scope of the present work. It should be noted that direct photoionisation of Mn^4+^ ions requires significantly higher energy, typically achieved at wavelengths shorter than 300 nm.

The integrated TSL intensities of the sol-gel powders are plotted against annealing temperature in Figure 9a. All five compositions show the same trend: the signal is strongest after treatment at 1100 °C and is very weak after treatment at 1500 °C. The decline accompanies sharper diffraction peaks and an increase in particle size from approximately 100–200 nm to 1–2 μm (Section 2.1). Higher-temperature treatment therefore appears to reduce the concentration of the accessible traps. PL and TSL consequently show different annealing optima: PL is strongest at 1450 °C, whereas TSL is strongest at 1100 °C. Among the samples annealed at 1100 °C, the unsubstituted LGYAO:Mn gives the strongest TSL, the Sr-containing ones are weaker, and the Ca-containing ones are the weakest, with Ca_0.10_ giving the smallest signal. The corresponding glow curves are collected in Figure 9e. The same ordering holds when the integrated TSL is normalised to the PL intensity of each sample, so it does not follow from the emission efficiency alone.

All samples release the stored charge in one broad band, with the underlying components appearing only as shoulders. Position and width both respond to substitution, but the two cations act differently. With Sr substitution, the temperature of maximum intensity shifts from 110 °C in the unsubstituted material to 100 °C at *x* = 0.05 and 70 °C at *x* = 0.10, with only slight broadening. Ca substitution at *x* = 0.05 produces the opposite shift: the maximum moves to 140 °C and the band narrows to a FWHM of approximately 70 °C. The Ca_0.10_ sample signal is too weak for its maximum and width to be determined reliably. No additional resolved maximum appears after substitution. The results are therefore compatible with a redistribution within a broad trap population rather than the appearance of a clearly separated trap family. This behaviour can be compared with *R*AlO_3_:Mn^4**+**^ perovskites containing one or two rare-earth cations, for which two separated glow peaks have been reported. These peaks have been assigned to intrinsic electron traps involving antisite defects together with oxygen and cation vacancies; in YAlO_3_ they were associated with trap depths of approximately 1.33 and 1.43 eV [26]. The comparison is qualitative because the temperature of a TSL maximum also depends on heating rate, frequency factor, retrapping and recombination kinetics. In the present samples, possible components are not resolved and appear instead as shoulders on a broad band. Variations in the local electrostatic and strain environment caused by mixed La/Gd/Y occupation of the A site could broaden the trap distribution [26]. Defect association and microstructure may also contribute, so the glow curves do not establish a unique microscopic trap model. The broad band is already present in the phase-pure sol-gel LGYAO sample annealed at 1100 °C. A secondary crystalline phase is therefore not required for its formation, although microstructural broadening cannot be excluded.

The solid-state samples differ from the sol-gel ones in the shape as well as in the intensity of their glow curves (Figure 9b–d). Their maximum lies at lower temperature and shifts only a little between LGYAO:Mn, LGYAO:Mn,Ca_0.05_ and LGYAO:Mn,Ca_0.10_, whereas the sol-gel maximum depends on the substituting cation. The YCaAl_3_O_7_ phase in the solid-state Ca-containing powders confirms partial Ca partitioning into a secondary phase. The similar peak positions may therefore indicate that the trap environment of the perovskite changes little with nominal Ca content. The integrated TSL intensity of the solid-state samples is also much higher (Figure 9f), exceeding that of the sol-gel sample by more than two orders of magnitude for LGYAO:Mn. Because the solid-state powders are multiphase, the difference reflects phase composition and microstructure as well as trap population and filling.

The composite-defect model proposed for *R*AlO_3_ hosts provides one possible way to interpret the substitution dependence. In this model, electron traps involve associated oxygen vacancies, antisite defects and rare-earth vacancies [26]. If Ca^2+^ or Sr^2**+**^ occupies a rare-earth site, its negative effective charge can compensate Mn^4+^ on an Al^3+^ site and alter the equilibrium concentrations of other compensating defects. The observed decrease in TSL may therefore arise from a reduction in the number or filling efficiency of the relevant trapping complexes. The larger Ca effect may be related to differences in incorporation and phase partitioning. The solid-state samples also show a marked decrease in integrated TSL after Ca addition, although their peak positions change little. Because YCaAl_3_O_7_ is present in these samples, this decrease cannot be assigned solely to Ca substitution at the perovskite A site.

### 2.7. Persistent Luminescence Behaviour

**Figure 10:**
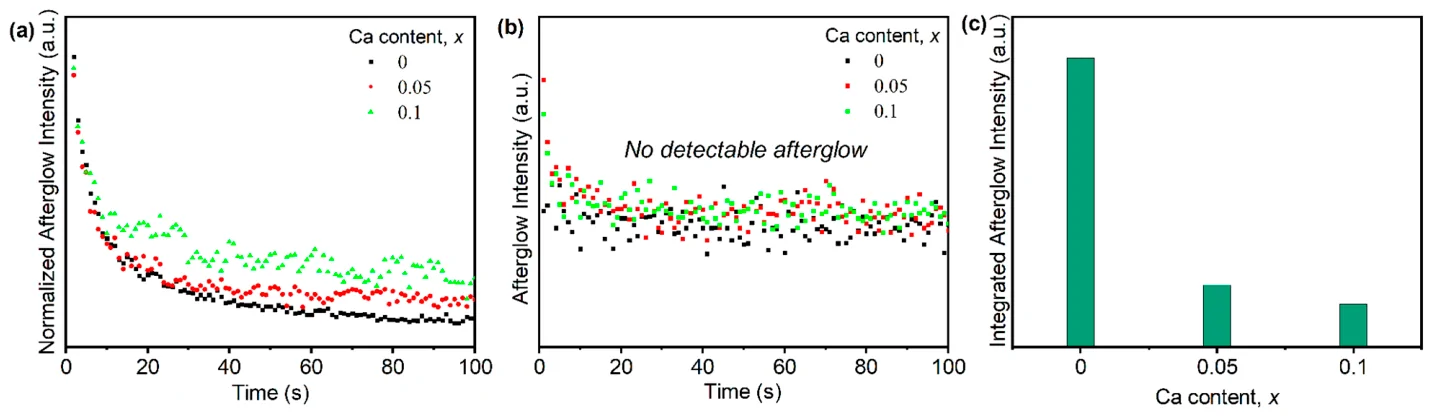
Persistent luminescence behaviour of Mn^4+^-doped medium-entropy aluminate samples recorded after 20 s excitation at 300 nm. (**a**) Normalised decay curves of the solid-state samples, showing the relative decay behaviour. (**b**) Raw afterglow signal of the sol-gel-derived samples, showing no detectable persistent luminescence under the applied conditions. (**c**) Integrated afterglow intensity of the solid-state samples calculated over the same time range. The unsubstituted LGYAO sample shows the strongest afterglow response, whereas Ca^2+^ substitution decreases the persistent luminescence intensity.

Persistent luminescence was recorded for 100 s following 20 s of excitation at 300 nm, and an afterglow was found only in the solid-state samples. Their normalised decay curves are shown in Figure 10a., where the signal falls steeply over the first 10 to 15 s and then declines slowly to the end of the recording. The sol-gel powders gave nothing comparable: as Figure 10b. shows, a weak signal persists for a few seconds after the excitation is switched off, after which the traces scatter about the background with no systematic decay. Within the detection limit of the experiment these samples show no persistent luminescence.

The TSL results help to explain the difference in afterglow behaviour. Persistent luminescence at room temperature requires a sufficient population of filled traps with release rates appropriate for the measurement time. The integrated TSL intensity of the sol-gel powders is more than two orders of magnitude lower than that of the solid-state samples, indicating that considerably less trapped charge is available for subsequent emission. Their glow maxima lie between 70 and 140 °C (Section 2.6), but the peak temperature alone cannot be used to determine the trap lifetime at room temperature. The absence of a measurable decay therefore shows that any charge released from the sol-gel samples during the 100 s recording produces a signal below the detection limit. The solid-state samples show a much larger TSL signal and a greater contribution on the low-temperature side of the glow band. This combination is consistent with the release of trapped charge near room temperature and with the afterglow observed in Figure 10a. The afterglow also depends on composition. As shown in Figure 10c, unsubstituted LGYAO:Mn has the highest integrated afterglow intensity, whereas both Ca-substituted samples give less than half of this value and differ only slightly from one another. The presence of YCaAl_3_O_7_ shows that some Ca is incorporated into a secondary phase. Increasing the nominal Ca content from *x* = 0.05 to 0.10 may therefore have only a limited effect on the Ca content of the perovskite, although this cannot be determined from XRD alone. Under the same excitation and measurement conditions, the integrated TSL and afterglow intensities follow the same order. This suggests that the amount of stored charge is an important factor controlling the afterglow intensity.

After approximately 20 s, the normalised decay curves of the Ca-substituted samples lie above that of the unsubstituted sample (Figure 10a). Since each curve is divided by its own maximum, this shows that the Ca-containing samples decay more slowly relative to their initial intensities; it does not mean that their absolute afterglow is stronger. Their total afterglow output remains lower, as shown in Figure 10c. The similar TSL peak positions of the three solid-state samples (Figure 9b–d) show that Ca substitution has little effect on the temperature of maximum charge release, but they do not establish identical trap depths or trap distributions. No afterglow was detected after excitation at 490 nm, in agreement with the TSL measurements. Under the conditions used here, excitation at 300 nm produces a measurable trapped-charge population.

## 3. Experimental Methods

### Sample and Experiment

Medium-entropy aluminate perovskite phosphors with the nominal composition (La_1/3_Gd_1/3_Y_1/3_)_1−*x*_*M_x_*AlO_3−*δ*_:0.1%Mn^4+^, where *x* = 0, 0.05 and 0.1 and *M* is Ca or Sr, were prepared by two routes: sol-gel and conventional solid-state.

In the sol-gel route, metal precursor solutions were obtained by dissolving the corresponding nitrates in distilled water or by dissolving metal oxides or carbonates in concentrated HNO_3_ (65 wt.%, >99.9%, Sfera Sim LLC, Lviv, Ukraine). The following reagents were used as precursor materials: Al(NO_3_)_3_∙9H_2_O (99.999%, ThermoFisher Scientific, Waltham, MA, USA), La(NO_3_)_3_∙6H_2_O (99.9%, ThermoFisher Scientific), Y(NO_3_)_3_∙6H_2_O (99.8%, Merck Group, Saint Louis, MO, USA), Mn(CH_3_COO)_2_∙4H_2_O (>99%, Merck Group), Ca(NO_3_)_2_∙4H_2_O (>99%, Merck Group), SrCO_3_ (99,9%, Merck Group), Gd_2_O_3_ (99.9%, ThermoFisher Scientific).

Calculated aliquots of metal nitrate solutions were mixed with citric acid (Sfera Sim LLC, pharmaceutical grade, >99%), which served as a complexing agent, and ethylene glycol (Sfera Sim LLC, pharmaceutical grade, >99%) to promote polyesterification and the formation of a three-dimensional polymeric network. Homogeneous solutions were heated at 80 °C under continuous stirring until gelation occurred. The obtained gels were subsequently dried at 105 °C to remove residual water and volatile components.

In the solid-state route, the powders of corresponding metal oxides were weighed according to the required composition, mixed, and ground in an agate mortar to homogenise the mixture. The mixtures were then synthesised in two steps at 1300 °C for 5 h and 1500 °C for 5 h, with intermediate grinding between the steps. A part of the samples was subsequently annealed at 1600 °C and used for comparison.

The following reagents were used as precursor materials for solid-state synthesis: La_2_O_3_ (99.999%, Johnson Matthey Catalysts GmbH, Redwitz an der Rodach, Germany), Gd_2_O_3_ (99.99%, Fox-Chemicals GmbH, Pfinztal, Germany), Y_2_O_3_ (99.99%, Johnson Matthey Catalysts GmbH), Al_2_O_3_ (99.99%, ThermoFisher Scientific), MnO_2_ (99.997%, ThermoFisher Scientific) and CaCO_3_ (99.99%, ThermoFisher Scientific).

Sample codes, nominal compositions and heat treatment temperatures of the (La_1/3_Gd_1/3_Y_1/3_)_1−*x*_*M*_*x*_AlO_3−*δ*_:0.1%Mn^4+^ materials prepared by two experimental techniques are presented in Table 3.

Thermal analysis (DTA/TG) of sol-gel derived precursor’s for a synthesis of (La_1_/_3_Gd_1_/_3_Y_1_/_3_)_1−*x*_*M_x_*AlO_3−*δ*_ perovskites was performed using a Linseis STA PT 1600 thermoanalyser (Linseis Messgeräte GmbH, Selb, Germany) in the temperature range between room temperature (RT) and 1500 °C with a heating rate of 10 °C/min. Phase and structural characterisation of the materials was carried out by X-ray diffraction (XRD) technique using an Aeris powder diffractometer (Malvern Panalytical, Worcestershire, UK) operating with Cu anode tube (λ = 1.54185 Å). Elemental composition of the samples and powder morphology were examined by scanning electron microscopy (SEM) coupled with energy-dispersive X-ray spectroscopy (EDS) by using an Apreo 2C LoVac microscope (Thermo Fisher Scientific) equipped with an UltraDry EDS detector featuring a 100 mm^2^ active area. To ensure statistical reliability, the EDX spectrum was collected over a prolonged total acquisition time of 500 s at an accelerating voltage of 20 keV, resulting in 13.05∙106 total counts with an average count rate of ~25,700 cps.

Photoluminescence (PL) emission and photoluminescence excitation (PLE) spectra were measured on a Horiba/Jobin–Yvon Fluorolog-3 spectrofluorometer (HORIBA Jobin Yvon IBH Ltd., Glasgow, UK) with a 450 W continuous-wave xenon lamp. The signal was detected by a Hamamatsu R928P photomultiplier working in photon-counting mode. All PL spectra were corrected for the spectral sensitivity of the detection system. Afterglow decay curves and thermally stimulated luminescence (TSL) glow curves were recorded on the same Fluorolog-3 setup in the real-time decay mode. The TSL measurements were made from 0 to 300 °C in a Linkam THMS600 heating stage (Linkam Scientific Instruments Ltd., Salfords, UK) at a constant heating rate of 1 °C/s. The luminescence decay kinetics were measured using the same Fluorolog-3 spectrofluorometer with the excitation light modulated by a mechanical chopper. The spectroscopic measurements in the temperature range 4.4–330 K were carried out in a Jenis continuous-flow liquid helium cryostat using a Lake Shore 331 temperature controller (Lake Shore Cryotronics, Westerville, OH, USA).

## 4. Conclusions

Mn^4+^-activated medium-entropy aluminate perovskites (La_1/3_Gd_1/3_Y_1/3_)_1−*x*_*M_x_*AlO_3−*δ*_:0.1%Mn^+4^ (*M* = Ca^2+^ or Sr^2+^) were prepared by a sol-gel and a solid-state route. Phase-pure perovskite was obtained only by the sol-gel route. The unsubstituted composition formed a single-phase perovskite at 1000 and 1100 °C, whereas a small amount of garnet phase appeared after annealing at 1450 and 1500 °C. Calcium-substituted materials form as phase-pure perovskite products at 1000 °C and remain single-phase up to a maximum temperature of 1500 °C. In the case of Sr-containing samples, precipitation of the minor amount of tetragonal phase RESrAl_3_O_7_ indicates that solubility limit of strontium in the medium-entropy perovskite (La_1/3_Gd_1/3_Y_1/3_)_1−*x*_Sr_*x*_AlO_3−*δ*_ is less than *x* = 0.1. Rietveld refinement of the structures of three selected materials confirms the aliovalent substitution of RE’s by alkaline-earth metal cations Ca^2+^ and Sr^3+^ in the medium-entropy perovskite (La_1/3_Gd_1/3_Y_1/3_)AlO_3_, resulting in the formation of oxygen-deficient structures. The solid-state obtained products are multiphase, containing in addition to the main perovskite phase, the impurities of garnet phases and RECaAl_3_O_7_ phase in case of the Ca-containing material.

All samples showed the ^2^E_g_ → ^4^A_2g_ emission near 700 nm, assigned to Mn^4+^ on the octahedral aluminium site. Its nearly unchanged position and profile indicate the same dominant octahedral Mn^4+^ centre throughout the series. The low-temperature PL spectra showed no detectable emission attributable to the garnet phase. The PL lifetime decreased with increasing annealing temperature, suggesting increased non-radiative relaxation that coincides with the increase in the impurity phase. In the sol-gel series, PL was strongest after annealing at 1450 °C, whereas TSL was strongest at 1100 °C. Ca^2+^ and Sr^2+^ substitution reduced both signals. The broad TSL band may reflect a distribution of trap environments associated with mixed La/Gd/Y occupation, although other structural defects may also contribute. For matched compositions, the solid-state samples showed stronger PL and much higher TSL intensity than the sol-gel samples, and afterglow was detected only in the solid-state series. The sol-gel route is therefore preferable for obtaining phase-pure perovskite, whereas the solid-state route gives the stronger persistent-luminescence response under the conditions studied.

## Figures and Tables

**Table 1:** Lattice parameters, fractional coordinates and displacement parameters of atoms in (La_1/3_Gd_1/3_Y_1/3_)AlO_3_, (La_1/3_Gd_1/3_Y_1/3_)_0.9_Ca_0.1_AlO_3−*δ*_ and (La_1/3_Gd_1/3_Y_1/3_)_0.9_Sr_0.1_AlO_3−*δ*_ structures.

Unit Cell Dimensions, Å, Å^3^	Atoms, Sites	x/a	y/b	z/c	Biso/eq, Å^2^	Occupancy *
(La_1/3_Gd_1/3_Y_1/3_)AlO_3_, 1100 °C; *R*_I_ = 0.0411; *R*_P_ = 0.0838						
*a* = 5.2898(4)	RE, 4*c*	0.9942(7)	0.0261(2)	¼	1.04(2)	* La^3+^, Gd^3+^, Y^3+^
*b* = 5.3105(5)	Al, 4*b*	0	½	0	0.89(6)	0.993(6) Al^3+^ + 0.007(6) Mn^4+^
*c* = 7.4812(7)	O1, 4*c*	0.067(2)	0.488(2)	¼	1.15(15)	0.99(2) O^2−^
*V* = 210.16(6)	O2, 8*d*	0.722(3)	0.283(3)	0.030(2)	1.15(15)	1.00(2) O^2−^
(La_1/3_Gd_1/3_Y_1/3_)_0.9_Ca_0.1_AlO_3−*δ*_, 1500 °C; *R*_I_ = 0.0298; *R*_P_ = 0.0764						
*a* = 5.2918(3)	RE/Ca, 4*c*	0.9959(9)	0.0164(3)	¼	1.33(3)	* La^3+^, Gd^3+^, Y^3+^, Ca^2+^
*b* = 5.2958(3)	Al, 4*b*	0	½	0	0.74(6)	0.992(7) Al^3+^ + 0.008(7) Mn^4+^
*c* = 7.4792(3)	O1, 4*c*	0.059(6)	0.496(3)	¼	1.8(2)	0.97(7) O^2−^
*V* = 209.60(3)	O2, 8*d*	0.742(5)	0.274(4)	0.030(3)	1.8(2)	1.00(4) O^2−^
(La_1/3_Gd_1/3_Y_1/3_)_0.9_Sr_0.1_AlO_3−*δ*_, 1500 °C (96.4 wt.%); *R*_I_ = 0.02221; *R*_P_ = 0.1438						
*a* = 5.2941(9)	RE/Sr, 4*c*	0.9915(3)	0.0194(2)	¼	0.74(3)	* La^3+^, Gd^3+^, Y^3+^, Sr^2+^
*b* = 5.2952(8)	Al, 4*b*	0	½	0	0.67(6)	0.993(7) Al^3+^ + 0.007(7) Mn^4+^
*c* = 7.5047(6)	O1, 4*c*	0.045(3)	0.494(2)	¼	1.1(3)	1.02(2) O^2−^
*V* = 210.38(8)	O2, 8*d*	0.709(4)	0.277(4)	0.025(2)	1.1(3)	0.96(1) O^2−^

* fixed according to nominal compositions.

**Table 2:** Photoluminescence lifetimes of LGYAO:Mn samples measured at 4.4 K and room temperature (RT), derived from single-exponential fits of the decay curves for different synthesis methods and annealing temperatures.

Synthesis Method	Annealing Temperature	Lifetime (τ) at 4.4 K, ms	Lifetime (τ) at RT, ms
Sol-gel	1100 °C	4.1	3.0
Sol-gel	1450 °C	3.2	2.2
Solid-state	1500 °C	3.0	2.1

**Table 3:** Sample codes, compositions, heat treatment temperatures and synthesis routes of the (La_1/3_Gd_1/3_Y_1/3_)_1−*x*_*M*_*x*_AlO_3−*δ*_:0.1%Mn^4+^ phosphors (*M* = Ca, Sr).

Code	Nominal Composition	Substitution	Heat Treatment	Synthesis Route
LGYAO:Mn	(La_1/3_Gd_1/3_Y_1/3_)AlO_3_:0.1% Mn^4+^	*x* = 0	800, 900, 1000, 1100, 1450, 1500 °C	Sol-gel
LGYAO:Mn,Ca_0.05_	(La_1/3_Gd_1/3_Y_1/3_)_0.95_Ca_0.05_AlO_3−*δ*_:0.1% Mn^4+^	*x* = 0.05, *M* = Ca	800, 900, 1000, 1100, 1450, 1500 °C	Sol-gel
LGYAO:Mn,Ca_0.10_	(La_1/3_Gd_1/3_Y_1/3_)_0.90_Ca_0.10_AlO_3−*δ*_:0.1% Mn^4+^	*x* = 0.10, *M* = Ca	800, 900, 1000, 1100, 1450, 1500 °C	Sol-gel
LGYAO:Mn,Sr_0.05_	(La_1/3_Gd_1/3_Y_1/3_)_0.95_Sr_0.05_AlO_3−_*_δ_*:0.1% Mn^4+^	*x* = 0.05, *M* = Sr	800, 900, 1000, 1100, 1450, 1500 °C	Sol-gel
LGYAO:Mn,Sr_0.10_	(La_1/3_Gd_1/3_Y_1/3_)_0.90_Sr_0.10_AlO_3−_*_δ_*:0.1% Mn^4+^	*x* = 0.10, *M* = Sr	800, 900, 1000, 1100, 1450, 1500 °C	Sol-gel
LGYAO:Mn	(La_1/3_Gd_1/3_Y_1/3_)AlO_3_:0.1% Mn^4+^	*x* = 0	1500, 1600 °C	Solid-state
LGYAO:Mn,Ca_0.05_	(La_1/3_Gd_1/3_Y_1/3_)_0.95_Ca_0.05_AlO_3−*δ*_:0.1% Mn^4+^	*x* = 0.05, *M* = Ca	1500, 1600 °C	Solid-state
LGYAO:Mn,Ca_0.10_	(La_1/3_Gd_1/3_Y_1/3_)_0.90_Ca_0.10_AlO_3−*δ*_:0.1% Mn^4+^	*x* = 0.10, *M* = Ca	1500, 1600 °C	Solid-state

## Data Availability

The data supporting the findings of this study are available from the corresponding author upon reasonable request.

## References

[1] Tsai M.-H., Yeh J.-W. (2014). High-Entropy Alloys: A Critical Review. Mater. Res. Lett..

[2] Yeh J.-W., Chen S.-K., Lin S.-J., Gan J.-Y., Chin T.-S., Shun T.-T., Tsau C.-H., Chang S.-Y. (2004). Nanostructured High-Entropy Alloys with Multiple Principal Elements: Novel Alloy Design Concepts and Outcomes. Adv. Eng. Mater..

[3] Miracle D.B., Senkov O.N. (2017). A Critical Review of High Entropy Alloys and Related Concepts. Acta Mater..

[4] Rost C.M., Sachet E., Borman T., Moballegh A., Dickey E.C., Hou D., Jones J.L., Curtarolo S., Maria J.-P. (2015). Entropy-Stabilized Oxides. Nat. Commun..

[5] Oses C., Toher C., Curtarolo S. (2020). High-Entropy Ceramics. Nat. Rev. Mater..

[6] Xiang H., Xing Y., Dai F., Wang H., Su L., Miao L., Zhang G., Wang Y., Qi X., Yao L. (2021). High-Entropy Ceramics: Present Status, Challenges, and a Look Forward. J. Adv. Ceram..

[7] Brahlek M., Gazda M., Keppens V., Mazza A.R., McCormack S.J., Mielewczyk-Gryń A., Musico B., Page K., Rost C.M., Sinnott S.B. (2022). What Is in a Name: Defining “High Entropy” Oxides. APL Mater..

[8] Chen K., Ma J., Wang H., Li C., An L. (2021). Entropy-Stabilized Oxides with Medium Configurational Entropy. Ceram. Int..

[9] Wright A.J., Luo J. (2020). A Step Forward from High-Entropy Ceramics to Compositionally Complex Ceramics: A New Perspective. J. Mater. Sci..

[10] Dippo O.F., Vecchio K.S. (2021). A Universal Configurational Entropy Metric for High-Entropy Materials. Scr. Mater..

[11] Witte R., Sarkar A., Kruk R., Eggert B., Brand R.A., Wende H., Hahn H. (2019). High-Entropy Oxides: An Emerging Prospect for Magnetic Rare-Earth Transition Metal Perovskites. Phys. Rev. Mater..

[12] Nguyen T.X., Liao Y., Lin C., Su Y., Ting J. (2021). Advanced High Entropy Perovskite Oxide Electrocatalyst for Oxygen Evolution Reaction. Adv. Funct. Mater..

[13] Ning Y., Pu Y., Wu C., Chen Z., Zhang X., Zhang L., Wang B. (2024). Design Strategy of High-Entropy Perovskite Energy-Storage Ceramics: A Review. J. Eur. Ceram. Soc..

[14] Gazda M., Miruszewski T., Jaworski D., Mielewczyk-Gryń A., Skubida W., Wachowski S., Winiarz P., Dzierzgowski K., Łapiński M., Szpunar I. (2020). Novel Class of Proton Conducting Materials—High Entropy Oxides. ACS Mater. Lett..

[15] Zhao Z., Rehder L., Steinbach F., Feldhoff A. (2022). High-Entropy Perovskites Pr1−xSrx(Cr,Mn,Fe,Co,Ni)O3−δ (x = 0–0.5): Synthesis and Oxygen Permeation Properties. Membranes.

[16] Shen L., Du Z., Zhang Y., Dong X., Zhao H. (2021). Medium-Entropy Perovskites Sr(FeαTiβCoγMnζ)O3- as Promising Cathodes for Intermediate Temperature Solid Oxide Fuel Cell. Appl. Catal. B Environ..

[17] Ning Y., Pu Y., Wu C., Zhang J., Zhang X., Shang Y. (2023). Achieving High Energy Storage Performance below 200 kV/Cm in BaTiO3-Based Medium-Entropy Ceramics. Ceram. Int..

[18] Li X., Shi C., Zhang G., Zheng G., Huang Z., Shen X., Zhou J., Chen T., Wang S. (2023). A Medium-Entropy Perovskite Oxide La0.7Sr0.3Co0.25Fe0.25Ni0.25Mn0.25O3-δ as Intermediate Temperature Solid Oxide Fuel Cells Cathode Material. Ceram. Int..

[19] Su L., Huyan H., Sarkar A., Gao W., Yan X., Addiego C., Kruk R., Hahn H., Pan X. (2022). Direct Observation of Elemental Fluctuation and Oxygen Octahedral Distortion-Dependent Charge Distribution in High Entropy Oxides. Nat. Commun..

[20] Barber J.P., Deary W.J., Titus A.N., Bejger G.R., Almishal S.S.I., Rost C.M. (2025). Disorder by Design: Unveiling Local Structure and Functional Insights in High Entropy Oxides. Mater. Horiz..

[21] He Q., Meng D., Ye W., Han Y., Chen K., Nie J., Zhu Z., Zhou S. (2025). A Multifunctional High-Entropy Perovskite Ceramics with Enhanced Energy Storage and Photoluminescence Properties. Energy Technol..

[22] Han Y., Wang S., Liu H., Shi L., Liu C., Shi X., Yang S., Tang M., Zhao L., Zhang Z. (2020). Far Red-Emitting Double Perovskite Phosphors Ca2(1-x)Sr2xGdSbO6:Mn4+: Luminescence Improvement Based on Composition Modulation. Ceram. Int..

[23] Zhou Q., Dolgov L., Srivastava A.M., Zhou L., Wang Z., Shi J., Dramićanin M.D., Brik M.G., Wu M. (2018). Mn2+ and Mn4+ Red Phosphors: Synthesis, Luminescence and Applications in WLEDs. A Review. J. Mater. Chem. C.

[24] Adachi S. (2020). Review—Mn4+ -Activated Red and Deep Red-Emitting Phosphors. ECS J. Solid State Sci. Technol..

[25] Sun Q., Wang S., Devakumar B., Li B., Sun L., Liang J., Chen D., Huang X. (2018). Novel Far-Red-Emitting SrGdAlO4:Mn4+ Phosphors with Excellent Responsiveness to Phytochrome PFR for Plant Growth Lighting. RSC Adv..

[26] Zhydachevskyy Y., Hizhnyi Y., Nedilko S.G., Kudryavtseva I., Pankratov V., Stasiv V., Vasylechko L., Sugak D., Lushchik A., Berkowski M. (2021). Band Gap Engineering and Trap Depths of Intrinsic Point Defects in RAlO3 (R = Y, La, Gd, Yb, Lu) Perovskites. J. Phys. Chem. C.

[27] Vasylechko L., Senyshyn A., Bismayer U., Gschneidner K.A., Bünzli J.C.G., Pecharsky V.K. (2009). Chapter 242 Perovskite-type aluminates and gallates. Handbook on the Physics and Chemistry of Rare Earths.

[28] Ueda J. (2021). How to Design and Analyze Persistent Phosphors?. Bull. Chem. Soc. Jpn..

[29] Srivastava A.M., Brik M.G. (2013). Crystal Field Studies of the Mn4+ Energy Levels in the Perovskite, LaAlO3. Opt. Mater..

[30] Noginov M.A., Loutts G.B. (1999). Spectroscopic Studies of Mn^4+ Ions in Yttrium Orthoaluminate. J. Opt. Soc. Am. B.

[31] Du J., De Clercq O.Q., Korthout K., Poelman D. (2017). LaAlO3:Mn4+ as Near-Infrared Emitting Persistent Luminescence Phosphor for Medical Imaging: A Charge Compensation Study. Materials.

[32] Du J., De Clercq O.Q., Poelman D. (2018). Thermoluminescence and Near-Infrared Persistent Luminescence in LaAlO3:Mn4+,R (R= Na+, Ca2+, Sr2+, Ba2+) Ceramics. Ceram. Int..

[33] Linderälv C., Åberg D., Erhart P. (2021). Luminescence Quenching via Deep Defect States: A Recombination Pathway via Oxygen Vacancies in Ce-Doped YAG. Chem. Mater..

[34] Padole A.S., Singar V., Ratnawat P., Dabkara A., Kar D.P., Jain N., Gupta M., Sagdeo A., Sagdeo P.R. (2025). Structural and Optical Characterization of Pure and Lanthanum Substituted Nd 1– x La x AlO3 Prepared by Sol-Gel Method. ChemistryOpen.

[35] Vasylechko L., Hreb V., Stadnik V., Lutsyuk I., Hirskyi Y., Buryy O., Zhydachevskyy Y. (2026). Crystal Structure and Thermal Expansion Behaviour of High-Entropy Perovskite (Y0.2Nd0.2Sm0.2Eu0.2Er0.2)AlO3. Sci. Rep..

[36] Akselrud L., Grin Y. (2014). WinCSD: Software Package for Crystallographic Calculations (Version 4). J. Appl. Crystallogr..

[37] Skakle J.M.S., Herd R. (1999). Crystal Chemistry of ( RE, A )2 M3 O7 Compounds (RE=Y, Lanthanide; A=Ba, Sr, Ca; M=Al, Ga). Powder Diffr..

[38] Shannon R.D. (1976). Revised Effective Ionic Radii and Systematic Studies of Interatomic Distances in Halides and Chalcogenides. Acta Cryst. A.

[39] Zhang H., Ding Q., Chen F., Miao Y., Yu D., Zhang D. (2024). Luminescent Properties of Mn^4+^ Impurity Center-Induced Mg_7_Ga_2_GeO_12_:Tb^3+^ Multicolor Phosphor. Chin. J. Lumin..

[40] Loutts G.B., Warren M., Taylor L., Rakhimov R.R., Ries H.R., Miller G., Noginov M.A., Curley M., Noginova N., Kukhtarev N. (1998). Manganese-Doped Yttrium Orthoaluminate: A Potential Material for Holographic Recording and Data Storage. Phys. Rev. B.

[41] Zhydachevskii Y., Galanciak D., Kobyakov S., Berkowski M., Kamińska A., Suchocki A., Zakharko Y., Durygin A. (2006). Photoluminescence Studies of Mn4+ Ions in YAlO3 Crystals at Ambient and High Pressure. J. Phys. Condens. Matter.

[42] Przybylińska H., Zhydachevskyy Y., Grochot A., Wołoś A., Stasiv V., Glowacki M., Kaminska A., Ubizskii S., Berkowski M., Suchocki A. (2022). Electron Paramagnetic Resonance and Optical Studies of Thermoluminescence Processes in Mn-Doped YAlO3 Single Crystals. J. Phys. Chem. C.

